# The inherent uncertainty of temporal networks is a true challenge for control

**DOI:** 10.1038/s41598-021-86059-8

**Published:** 2021-03-26

**Authors:** Pietro De Lellis, Anna Di Meglio, Franco Garofalo, Francesco Lo Iudice

**Affiliations:** Department of Electrical Engineering and Information Technology, University of Naples Federico II, Via Claudio 21, 80125 Naples, Italy

**Keywords:** Complex networks, Electrical and electronic engineering, Physics, Statistical physics, thermodynamics and nonlinear dynamics

## Abstract

Recently, it has been suggested that network temporality can be exploited to substantially reduce the energy required to control complex networks. This somewhat counterintuitive finding was explained through an evocative example of the advantage of temporal networks: when navigating a sailboat, we raise the sails when the wind helps us while lowering them when it works against us. Unfortunately, controlling complex networks inherits a further analogy with navigating a sailboat: having to face the inherent uncertainty of future winds. We rarely, if ever, have deterministic knowledge of the evolution of the network we want to control. Here, our challenge is to exploit the potential advantages of temporality when only a probabilistic description of the future is available. We prove that, in this more realistic setting, exploiting temporality is no more a panacea for network control, but rather an asset of a wider toolbox made available by the scientific community. One that can indeed turn out useful, provided that the temporality of the network structure matches the intrinsic time scales of the nodes we want to control.

## Introduction

Social networks^1,2^, animal groups^3^, power grids^4^, and metabolic networks^5^ are examples of large scale systems composed of several interacting entities. Whether we are interested in predicting the emergence of collective behavior, or whether we want to control these systems, the complex dynamical networks paradigm has proved to be the modeling tool of choice. Within this framework, the current state of the single entities, the *n* network nodes, is collected in a vector $x(t) = [x_1(t), \ x_2(t), \dots , x_n(t)]^T$, and the current value of the *p* external signals used to control the network behavior in a vector $u(t) = [u_1(t), \ u_2(t), \dots , u_p(t)]^T$. As the control signals only affect a limited number of the network nodes, the drivers, controlling complex networks entails exploiting the structure of the interconnections to indirectly affect the remainder of the nodes. Early work on network controllability, the ability to steer the vector *x*(*t*) towards any arbitrary state in finite time, hinted to the possibility of controlling real world complex systems by only leveraging a limited number of drivers^6–9^. However, this enthusiasm has been promptly tempered by the finding that reducing the number of drivers comes at the price of an exponential increase in the energy required to control a network^10,11^. When only few nodes are directly controlled, even numerically computing the control signal required to steer the network towards an arbitrary state becomes unfeasible^12^. Is there any workaround to this problem except increasing the number of drivers? The authors of Ref.^13^ suggested that many complex systems may possess natural mechanisms to avoid traveling along directions of the state space that would require an excessive amount of energy. More recently, Li and collaborators^14^ suggested that exploiting temporality, that is, network variability over time, could substantially reduce energy requirements without increasing the number of drivers. This finding, being counterintuitive, is intriguing, as we might expect that temporality might hinder our ability to control a network. However, this apparent contradiction is due to the fact that we humans often associate uncertainty to future variability. In the scenario considered in^14^, instead, there is variability, but without uncertainty, that is, the future evolution of the network is a priori known. In this ideal framework we would certainly feed energy to the network only when it is in a favorable configuration. In other words, we would act as an investor who sells her stocks only when they reach their peak values, while holding them otherwise. Unfortunately, real world investors cannot rely on knowledge of the future, but must forecast market peaks based on stochastic models, thus accepting the possibility of incurring into unpredicted losses. In the same spirit of the investors trusting technical analysis, we revisit the problem of controlling a temporal network in a stochastic setting, where only a probabilistic description of the network evolution is available. The control tools we develop allow us to uncover when the opportunities offered by temporality prevail over uncertainty on the future network evolution.

## Minimizing the expected control energy

A temporal network can be viewed as an ordered sequence of *m* static networks, from now on denoted as snapshots, sharing the same set of *n* nodes. The *k*-th snapshot, characterized by a weighted adjacency matrix $$A_k$$, describes the node interconnections in the interval $$[t_{k},t_{k+1})$$. The length of each interval $$\delta _k:=t_{k+1}-t_{k}$$ can be interpreted as a measure of the current network temporality. When $$\delta _k$$ is small, the network is experiencing a period of fast temporality, while when it is large, the temporality is slow. As in previous works^6,7,10,14,15^, we focus on temporal networks of linear systems

1 $$\begin{aligned} \dot{x}(t) = A_{k} x(t) + Bu(t), \qquad t\in [t_k, \ t_{k+1}), \quad k=0, \ldots , m-1. \end{aligned}$$

In Eq. (1), the matrix *B* identifies the set, equal for all snapshots, of *p* driver nodes that we directly influence through the control input *u* with the final goal of controlling the network as a whole. To highlight the fundamental distinction between our setting and previous work^14^, we give the definition of stochastic temporal network we refer to.

### **Definition 1**

Network (1) is a *stochastic temporal network* when, at any snapshot $k=0,\ldots ,m-1$, $A_k$ is drawn from a family ${\mathcal {F}}= \left\{ F_i \right\} _{i\in {\mathcal {I}}}$ of admissible adjacency matrices according to the realization of a scalar stochastic process $\sigma (k)\in {\mathcal {I}}$.

Namely, if at time $t_k$ we have $\sigma (k)={\bar{\sigma }}$, then $A_k = F_{{\bar{\sigma }}}$. Consistently, we will consider any observed sequence $A_k$, $k=0,\dots ,m-1$ as a realization of the stochastic process $A_{\sigma (k)}$. Therefore, the main difference with respect to the existing literature is in the *a priori* information available at any time $t\in [t_k,t_{k+1})$ for the control design. Indeed, while we still assume to have deterministic knowledge of the current realization $A_k$ of $A_{\sigma (k)}$, we can only rely on a probabilistic description of the future snapshots that will depend upon the distribution of the process $\sigma (i)$. In this stochastic scenario, does temporality still represent an advantage for network control?

To answer this question, we must first give a condition for controllability that suits this scenario. As the sequence of future snapshots is unknown *a priori*, guaranteeing that a temporal network is controllable implies selecting a *B* such that any possible realization of the pair $(A_k,B)$, $k=0,\dots , m-1$ is controllable. Under this assumption, to investigate whether temporality can mitigate the control effort, we developed a machinery inspired to stochastic optimal control^16^. This theoretical framework prescribes to find the signal *u*(*t*) that minimizes the *expected* energy required to drive the network from an initial state $x(t_0)$ to a final state $x(t_m)$, that is,

2a $$\begin{aligned}&\min _{u(t)} \mathop {\mathrm {E}}_{\varsigma _1}\left[ J(u(t)):=\int _{0}^{t_m}u(t)^Tu(t)\mathrm {d}t\right] , \end{aligned}$$

2b $$\begin{aligned}&\dot{x}(t) = A_{\sigma (k)} x(t) + Bu(t) \qquad t\in \ [t_k, \ t_{k+1}), \quad k=0, \ldots , m-1, \end{aligned}$$

2c $$\begin{aligned}&A_{\sigma (0)}=A_0, \ x(t_0)=x_0, \ x(t_{m})=x_{m}, \end{aligned}$$

where, in (2a), $\varsigma _k$ denotes the vector $\left[ \sigma (k),\ldots ,\sigma (m-1)\right]$, and

$$\begin{aligned} \mathop {\mathrm {E}}_{\varsigma _1}\left[ J(u)\right] =\int _{-\infty }^{+\infty }\cdots \int _{-\infty }^{+\infty }J(u)f_{\varsigma _1}(\varsigma _1)\mathrm {d}\sigma (1)\cdots \mathrm {d}\sigma (m-1), \end{aligned}$$

with $f_{\varsigma _1}$ being the joint probability distribution of the variables $\sigma (1),\ldots ,\sigma (m-1)$.

To solve the optimal control problem in Eq. (2), we must preliminarily note that in moving from $x(t_0)$ to $x(t_m)$ the network state will cross $m-1$ waypoints $x(t_k)$. It turns out that minimizing (2a) implies transitioning between any two consecutive waypoints with minimum energy. This can be achieved, in each snapshot, by means of the classic minimum energy control. Indeed, we can write

3 $$\begin{aligned} \mathop {\mathrm {E}}_{\varsigma _1}\left[ J(u)\right] =\mathop {\mathrm {E}}_{\varsigma _1}\left[ \sum _{k=0}^{m-1}J_k(u_k):= \sum _{k=0}^{m-1} \int _{t_k}^{t_{k+1}}u_k(t)^Tu_k(t)\mathrm {d}t\right] , \end{aligned}$$

where $$u_k(t)$$ is the restriction of *u*(*t*) to $$[t_k,t_{k+1})$$, for $$k=0,\ldots ,m-1$$. For given values of $$x(t_k)$$, $$x(t_{k+1})$$, and given the realization $$A_k$$ of $$A_{\sigma (k)}$$, the input $$u^*_k$$ minimizing $$J_k$$ is the well-known solution of the standard minimum energy control problem

4 $$\begin{aligned} \begin{aligned} \min _{u_k}&\, J_k(u_k)\\&\text {s.t.}\\ {\dot{x}}(t)&=A_{k}x(t)+Bu(t),\quad t\in [t_k,t_{k+1}),\\ x(t_k)&=x_k,\ x(t_{k+1})=x_{k+1}. \end{aligned} \end{aligned}$$

Namely, the optimal solution is

5 $$\begin{aligned} u^*_k(x_k,x_{k+1},t)= B^Te^{A^T_{k}(t_{k+1}-t)}W_{k}^{-1}\left( x_{k+1}-e^{A_{k}(t_{k+1}-t_k)}x_k\right) , \qquad t\in [t_k \ t_{k+1}), \end{aligned}$$

where

$$\begin{aligned} W_{k} = \int _{t_k}^{t_{k+1}} e^{{A_k}(t_{k+1}-\tau )}BB^Te^{A^T_{k}(t_{k+1}-\tau )}d\tau \end{aligned}$$

is the reachability gramian. For this well-known problem, the optimal value of the cost function is

$$\begin{aligned} \left( x_{k+1}-e^{\delta _kA_{k}}x_k\right) ^TW_k^{-1}\left( x_{k+1}-e^{\delta _kA_{k}}x_k\right) . \end{aligned}$$

Coming back to our problem in Eq. (2), noting that $J_k(u_k)\ge J_k(u^*_k)$ for all possible $x(t_k)$, $x(t_{k+1})$, and for any realization $A_k$ of $A_{\sigma _k}$ we can conclude that the structure of the solution of (2) is obtained by substituting $A_{\sigma (k)}$ to $A_k$ in Eq. (5). This implies that problem (2) can be viewed as a concatenation of different instances of problem (4) in each of which $x_k$ is given and $x_{k+1}$ is the actual decision variable. Accordingly, solving (2) becomes equivalent to selecting the optimal waypoints $x^*_1,\ldots ,x^*_{m-1}$. Therefore, the a priori control energy required in each snapshot is the stochastic variable

6 $$\begin{aligned} J_k(x_k,x_{k+1},\sigma (k))=\left( x_{k+1}-e^{\delta _kA_{\sigma (k)}}x_k\right) ^TW_k^{-1}\left( x_{k+1}-e^{\delta _kA_{\sigma (k)}}x_k\right) . \end{aligned}$$

and we can rewrite the minimum energy control problem as

7a $$\begin{aligned} \underset{{\begin{array}{c} x_1\\ \ldots \\ x_{m-1} \end{array}}}{\min} \mathop {\mathrm {E}}_{\varsigma _1}\left[ \sum _{k=0}^{m-1}J_k(x_k,x_{k+1},\sigma (k))\right] , \end{aligned}$$

7b $$\begin{aligned} A_{\sigma (0)}=A_0, \ x(t_0)=x_0, \ x(t_{m})=x_{m}, \end{aligned}$$

where, at each *k*, $A_{\sigma (k)}$ is known. The following theorem provides a recursive solution for computing the optimal waypoints.

### **Theorem 1**

*The solution of the optimal control problem* (7) *is given by*

8 $$\begin{aligned} x_k^*=P_k x_m+Q_k x_{k-1}, \quad k=1,\ldots ,m-1, \end{aligned}$$

*where*

9 $$\begin{aligned} \begin{aligned} P_k&=\left( W_{k-1}^{-1}+\sum _{i=k}^{m-1}\mathop {\mathrm {E}}_{\varsigma _k}\left[ {R_k^i}^T W_i^{-1}R_k^i\right] \right) ^{-1}\sum _{i=k}^{m-1}\mathop {\mathrm {E}}_{\varsigma _k}\left[ {R_k^i}^T W_i^{-1} F_k^i\right] ,\\ Q_k&=\left( W_{k-1}^{-1}+\sum _{i=k}^{m-1}\mathop {\mathrm {E}}_{\varsigma _k}\left[ {R_k^i}^T W_i^{-1}R_k^i\right] \right) ^{-1}W^{-1}_{k-1}e^{\delta _{k-1}A_{\sigma (k-1)}}, \end{aligned} \end{aligned}$$

*with*

10 $$\begin{aligned} \begin{aligned} R_k^i&=\left\{ \begin{matrix} e^{\delta _k A_{\sigma (k)}}-Q_{k+1}, &{}\,\ \ i=k,\\ \displaystyle R_{k+1}^i Q_{k+1}, &{}\,\ \ i> k, \end{matrix} \right. \quad k=0,\ldots ,m-2,\\ F_k^i&=\left\{ \begin{matrix} P_{k+1}, &{} i=k,\\ \displaystyle F_{k+1}^{i}-R_{k+1}^i P_{k+1}, &{} i> k, \end{matrix}\right. \quad k=0,\ldots ,m-2,\\ R_{m-1}^{m-1}&=e^{\delta _{m-1}A_{\sigma (m-1)}}, \quad \ \, \qquad F_{m-1}^{m-1}=I. \end{aligned} \end{aligned}$$

*Furthermore, the associated optimal cost is given by*

11 $$\begin{aligned} \mathop {\mathrm {E}}_{\varsigma _1}\left[ \sum _{i=0}^{m-1}\left( F_0^i x_m -R_0^i x_0\right) ^T W_i^{-1}\left( F_0^i x_m -R_0^i x_0\right) \right] . \end{aligned}$$

### *Proof*

See Supplementary Information, section S1.2. $\square$

**Figure 1:**
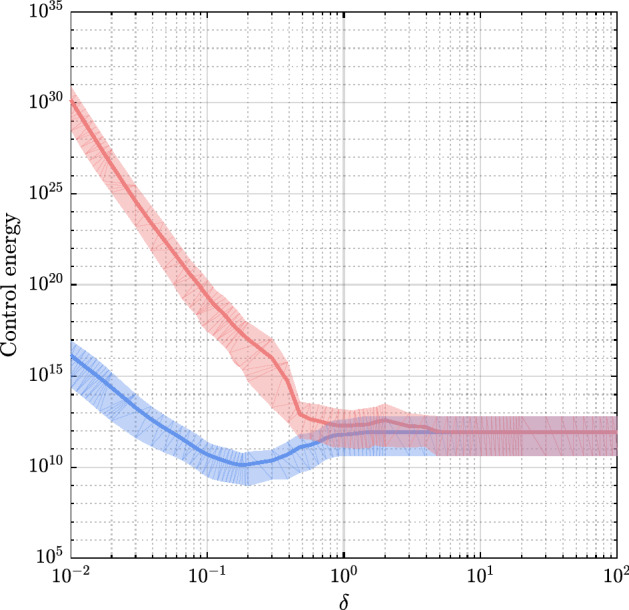
Control energy requirements in the deterministic (blue) and stochastic (red) scenario for the yeast *Saccharmoyces cerevisiae*. The solid lines are the minimum energies as a function of $\delta$ averaged over $10^3$ final states $x(t_m)$ selected on the unit hypersphere centered in the origin. The shaded areas are enclosed by the observed minimum and maximum energies. The minimum energy feedback control strategy is implemented both in the stochastic and in the deterministic scenario. The picture shows that in the stochastic scenario the control can be orders of magnitude more energetically demanding with respect to the deterministic scenario. The energy gap tends to vanish when $\delta$ increases, that is, when the temporality becomes slower.

The expression in (11), that is, the expected energy required to drive the temporal network from any $x(t_0)$ to any other $x(t_m)$ turns out to be a quadratic form. We leverage the developed machinery to compare the tasks of controlling a temporal network in the deterministic setting considered in Ref.^14^ and in our stochastic setting. An explicatory illustration of the effect of uncertainty is reported in Fig. 1, which depicts the energy required to control the same 83 node temporal network considered in Ref.^14^ and obtained by condensing the time-varying protein-protein binding interactions^17^ of the yeast Saccharmoyces Cerevisiae over 50 snapshots of equal length $\delta$. We have ensured each snapshot is asymptotically stable by adding self loops on the diagonal of each of the network adjacency matrices. Morover, we have selected 14 driver nodes so that each possible realization of the pair $(A_{\sigma (k)},B)$ is controllable. To allow a fair comparison between the stochastic and the deterministic setting, in both cases we take the sequence of snapshots directly from the data. Whereas in the stochastic case we assume this sequence is unknown and thus the waypoints must be computed online, in the deterministic case the sequence is known *a priori* allowing to compute the waypoints in advance. We find that the stochastic setting demands orders of magnitude more energy when the network temporality is fast. This difference reduces and asymptotically vanishes as the duration of each snapshot increases. Is this a general pattern? In the next section, we introduce the theoretical tools needed to answer this question.

## Expected energy for stochastic temporal and static networks

The previous section showed the striking impact of uncertainty, which seems to challenge the claim that network variability enhances our ability to control complex networks^14^. However, the observation was made with reference to one specific temporal network, i.e. a single realization of the process $\sigma (k)$, and for an arbitrarily selected set of target states $x_m$. To paint an unbiased picture of whether temporality can still be exploited to our advantage in this stochastic setting, we turn to synthetic networks to ensure independence from the specific sequence of snapshots and from the target state $x_m$. We consider networks of $N=100$ nodes, build a finite pool ${\mathcal {F}}$ of $|{\mathcal {I}}|=3$ network topologies, and select 10 driver nodes defining a matrix *B* that guarantees network controllability for any matrix in the pool (see Supplementary Information Section S3 for further details). We assess whether the advantage of temporality persists when the inherent presence of uncertainty is considered by comparing two scenarios. In the first one, we considered a stochastic temporal network as described in Definition 1 where the stochastic process $\sigma (k)$ is a sequence of independent uniformly distributed stochastic variables, and computed the *a priori* explected energy required to control it. This expected energy is then compared with the average energy required to control the network in a second scenario, in which we consider a static network whose topology has been extracted from ${\mathcal {F}}$ according to the uniformly distributed random variable $\sigma \in {\mathcal {I}}$. For the first scenario, from Eq. (11), the *a priori* expected energy required to control a stochastic temporal network can be computed as

12 $$\begin{aligned} J^*_{\mathrm {temporal}}=\mathop {\mathrm {E}}_{\sigma (0)}\left[ \mathop {\mathrm {E}}_{\varsigma _1}\left[ \sum _{i=0}^{m-1}\left( F_0^i x_m -R_0^i x_0\right) ^T W_i^{-1}\left( F_0^i x_m -R_0^i x_0\right) \right] \right] . \end{aligned}$$

Without loss of generality, we assume $x_0=0$, and denote

$$\begin{aligned} W_{\mathrm {exp}}^{-1}:= \mathop {\mathrm {E}}_{\varsigma _0}\left[ \sum _{i=0}^{m-1}F_0^{i\; T}\,W_i^{-1}F_0^i\right] \end{aligned}$$

the inverse of the *a priori* expected gramian over the entire time horizon $\left( t_0, t_m\right)$. Equation (12) can be then rewritten as

$$\begin{aligned} J^*_{\mathrm {temporal}}=x_m^T W_{\mathrm {exp}}^{-1}x_m. \end{aligned}$$

The trace of $W_{\mathrm {exp}}^{-1}$ represents the expected energy required to control a stochastic temporal network averaged over all possible target states on the unit hypersphere, and thus considering it ensures independence from a specific $x_m$. In the second scenario, the expected energy required to control a static network is

13 $$\begin{aligned} J^*_{\mathrm {static}}:=\mathop {\mathrm {E}}_{\sigma }\left[ \left( x_m-e^{(t_m-t_0)A_{\sigma }}x_{0}\right) ^TW_{\sigma }^{-1}\left( x_m-e^{(t_m-t_0)A_{\sigma }}x_{0}\right) \right] , \end{aligned}$$

where

14 $$\begin{aligned} W_{\sigma } = \int _{t_0}^{t_{m}} e^{A_{\sigma }(t_{m}-\tau )}BB^Te^{A_{\sigma }^T(t_{m}-\tau )}\mathrm {d}\tau . \end{aligned}$$

Hence, the trace of $\mathop {\mathrm {E}}_{\sigma }\left[ W_{\sigma } ^{-1} \right]$ provides a benchmark for the expected energy required to control a stochastic temporal network that is independent of the target state $x_m$. Notice that our choice is different from that made in^14^, where the benchmark was selected as the energy required to control the average network described by the matrix ${\bar{A}}=\mathop {\mathrm {E}}_{\sigma }\left[ A_{\sigma }\right]$, a choice that would yield the paradox of temporality being beneficial even when so slow to be considered negligible.

**Figure 2:**
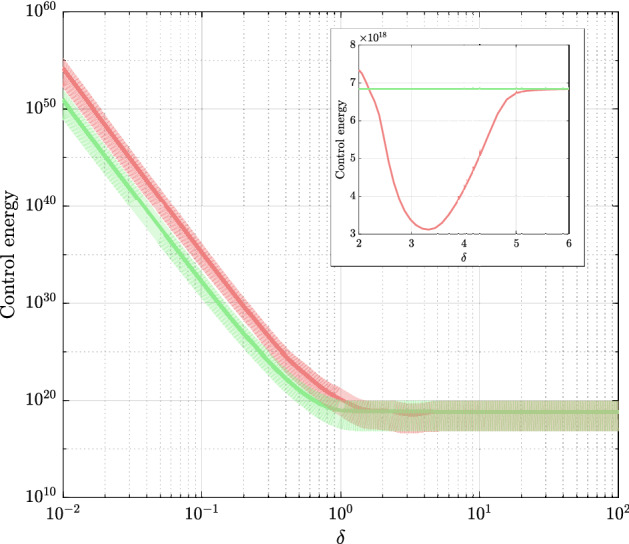
The advantage of temporal networks in the stochastic scenario. The solid lines represent the minimum expected energy averaged over all possible final states on the unit hypersphere centered in the origin (trace of $\mathop {\mathrm {E}}\left[ W_{\text{exp}} ^{-1} \right]$, in red) and the benchmark energy (trace of $\mathop {\mathrm {E}}\left[ W_{\sigma} ^{-1} \right]$, in green) as a function of $\delta =(t_m-t_0)/m$. The shaded areas are enclosed by the minimum and the maximum $J_{\text{temporal}^{*}}$ (in red) and $J_{\text{static}^{*}}$ (in green) observed over $10^{5}$ final states on the unit hypersphere. In the fast regime (i.e., for small $\delta$), uncertainty prevails over temporality with the expected energy required to control a temporal network being larger than the benchmark energy. As temporality vanishes (i.e., for large $\delta$), the energy difference becomes negligible. The advantage of temporality appears in the intermediate temporality regime and is shown in the inset.

**Figure 3:**
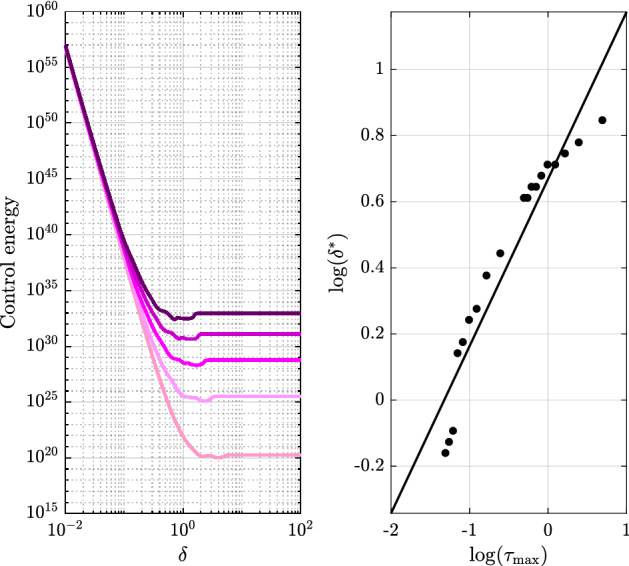
Network temporality and time scales. We consider 19 temporal networks with $n=100$ nodes over $m=3$ snapshots and differing only in the dominant time constant $\tau _{\max }$ that is chosen as a measure of the network time scale (see Supplementary Information section S2). The left panel shows the expected control energy averaged over all possible final states $x(t_m)$ on the unit hypersphere centered in the origin for 5 of the 19 networks. The plot illustrates that the network becomes more energetically demanding and that the minimum point of the energy shifts towards faster temporality regimes as $\tau _{\max }$ becomes smaller (i.e., as the curves become darker). The right panel highlights the relation between temporality and time scales, with the black dots representing $\log (\delta ^*)$ as a function of $\log (\tau _{\max })$ for each of the 19 networks. Specifically, the minimum point $\delta ^*$, numerically obtained, corresponds to the value of $\delta$ associated to the minimum expected control energy.

The results of the comparison between scenarios one and two are shown in Fig. 2. We find that in the fast temporality regime (with very small $\delta$) the expected energy required to control a temporal network can exceed by orders of magnitude that required by a static network. On the other hand, in the slow temporality regime, we observe that this difference becomes negligible. These empirical observations are supported by the theoretical analysis performed in Supplementary Information section S2. Our derivations provide a formal proof of the intuition that when the temporality is so fast that we do not have time to exploit it, the effect of uncertainty prevails. When instead the temporality is so slow that most of the energy fed to the network in order to reach a targeted waypoint is dissipated in the next snapshots, we rather wait for the last snapshot, thus treating a temporal network as if it were static. Our formal analysis clarifies that the paradoxical result reported in Ref.^14^ that temporality is advantageous even when so slow to be negligible was due to the use of an *ad hoc* static benchmark. Interestingly, we do find that there is a regime where temporality prevails on uncertainty (see the inset of Fig. 2). To delve into this regime, we should take into account that all real world systems that can be modeled as dynamical network are characterized by time scales. Those of digital communication networks^18^, for instance, are determined by the dynamical flow of the data packets, whereas those of epidemic processes^19^ depend on the specific infection taking place, and can range from few days to months^20^. For a linear network, and thus for each of our snapshots, the time scale is related to the eigenvalues of $A_k$. Our numerical results reveal that shifting the spectrum of the snapshots shifts the regime where the advantage of temporality appears (see Fig. 3). In other words, temporality prevails on uncertainty, provided it matches the time scale of the network we are trying to control. Our results prove to be robust to the removal of the hypothesis of independence of the stochastic process $\sigma (k)$, to variations of the size of the pool ${\mathcal {F}}$, and to variations of the number of snapshots *m* (see SI Section 4).

## Discussion

Coming back to our fundamental question, in real world systems, temporality comes hand in hand with uncertainty. Who can determinstically predict the future chemical reactions in a metabolic network, or the time instant at which a mobile device will activate? In this realistic scenario, is exploiting temporality the workaround to achieve the chimera we are chasing since 2011^6^, that is, controlling complex networks with a very limited number of driver nodes? Our results indicate this is not true, as we never experience that temporality yields orders of magnitude of energy reductions. However, is this a setback for the community working on network control? A careful analysis of our work shows that the answer is no. Rarely, if ever, we find a panacea for real-world problems, and network control proves to be no exception. More often, we develop assets that blended together yield substantial progress. Our findings show that the ability to exploit temporality is one of these assets, one that can allow halving energy requirements, provided it matches the time scale of the network we are trying to control. To put this advantage in perspective, in the aerospace industry, millions of dollars are spent to gain single digit advantages in fuel efficiency.

## Supplementary Information

Supplementary Information.
