# Supplementary material for: The inherent uncertainty of temporal networks is a true challenge for control
Source: Sci Rep. 2021 Mar 26;11:6977. doi: 10.1038/s41598-021-86059-8 (PMC7998000; doi:10.1038/s41598-021-86059-8)
Supplement: Supplementary file 1 — Supplementary Information. [file 41598_2021_86059_MOESM1_ESM.pdf]

The inherent uncertainty of temporal networks is a true challenge for control

Pietro DeLellis,[1] Anna Di Meglio,[1] Franco Garofalo,[1] Francesco Lo Iudice[*1]

[1]Department of Electrical Engineering and Information Technology, University of Naples Federico II, Via Claudio 21, Naples, 80125, Italy

[*]Corresponding author. E-mail: francesco.loiudice2@unina.it.

February 2, 2021

S1 Optimal control of stochastic temporal networks

S1.1 Stochastic temporal networks

Here, we refer to a class of temporal networks denoted interval graphs in [1], where the nodes are given, while the edges are active over a set of time intervals $\mathcal{T} = \{(t_0, t_1), \ldots, (t_{m-1}, t_m)\}$. Further, we consider nontrivial graphs with $n \geq 2$ nodes. A wide range of natural and artificial systems can be modeled as interval graphs. For instance, this is the case of ensembles of mobile agents, where proximity plays a relevant role in determining their interaction patterns: the edge between a pair of agents is only active in the time intervals when they have been sufficiently close. Other notable examples of systems where interval graphs modeling is required include computer networks where continuous network connectivity may lack [2], neuroscience [3], and finance [4], among the others.

In interval graphs, the network topology in $[t_k, t_{k+1})$ can be described by a (static) adjacency matrix A_k, $k = 0, \ldots, m-1$. As in our work we consider instances where the network topology is not known a priori, we introduce the *stochastic* interval graph, that is, an interval graph in which the adjacency matrices are independently drawn from a family $\mathcal{F} = \{A_i\}_{i \in \mathcal{I}}$ according to a given probability distribution. Hence, the sequence of adjacency matrices regulating the network topology becomes a stationary stochastic process. To simplify the notation in the remainder of this material, we model this by enforcing that at each time-instant the realization $A_k = A_{\sigma(k)} = [a_{ij}(\sigma(k))]_{i,j=1}^n$ depends on the value of the i.i.d. switching signal $\sigma(k)$.

Denoting by $x(t) \in \mathbb{R}^n$ the state of the network at time t, we then have the following stochastic temporal network

$$\dot{x}(t) = A_{\sigma(k)} x(t) + B u(t), \qquad t \in [t_k, t_{k+1}), \;\; k = 0, 1, 2, \ldots \tag{S1}$$

where $B \in \mathbb{R}^{n \times p}$ is the time-invariant input matrix that identifies the set of $p \leq n$ driver nodes which we directly influence through the control input $u(t)$. Similar to [5], we refer to the interval

$[t_k, t_{k+1})$ as the k-th snapshot. As we aim at controlling (S1) over a finite time interval $[t_0, t_m)$, we require that (A, B) is controllable for all $A \in \mathcal{F}$, which is necessary and sufficient for (S1) to be controllable for every possible realization of the stochastic interval graph.

S1.2 Minimum energy control

Theorem 1. *The solution of the optimal control problem (7) of the main manuscript is given by*

$$x_k^* = P_k x_m + Q_k x_{k-1}, \quad k = 1, \dots, m-1, \tag{S2}$$

where

$$\begin{aligned} P_k &= \left(W_{k-1}^{-1} + \sum_{i=k}^{m-1} \operatorname*{E}_{\varsigma_k} \left[{R_k^i}^T W_i^{-1} R_k^i \right] \right)^{-1} \sum_{i=k}^{m-1} \operatorname*{E}_{\varsigma_k} \left[{R_k^i}^T W_i^{-1} F_k^i \right], \\ Q_k &= \left(W_{k-1}^{-1} + \sum_{i=k}^{m-1} \operatorname*{E}_{\varsigma_k} \left[{R_k^i}^T W_i^{-1} R_k^i \right] \right)^{-1} W_{k-1}^{-1} e^{\delta_{k-1} A_{\sigma(k-1)}}, \end{aligned} \tag{S3}$$

with

$$\begin{aligned} R_k^i &= \begin{cases} e^{\delta_k A_{\sigma(k)}} - Q_{k+1}, & i = k, \\ R_{k+1}^i Q_{k+1}, & i > k, \end{cases} \qquad k = 0, \dots, m-2, \\ F_k^i &= \begin{cases} P_{k+1}, & i = k, \\ F_{k+1}^i - R_{k+1}^i P_{k+1}, & i > k, \end{cases} \qquad k = 0, \dots, m-2, \\ R_{m-1}^{m-1} &= e^{\delta_{m-1} A_{\sigma(m-1)}}, \qquad F_{m-1}^{m-1} = I. \end{aligned} \tag{S4}$$

Furthermore, the associated optimal cost is given by

$$J^* = \operatorname*{E}_{\varsigma_1} \left[\sum_{i=0}^{m-1} \left(F_0^i x_m - R_0^i x_0 \right)^T W_i^{-1} \left(F_0^i x_m - R_0^i x_0 \right) \right]. \tag{S5}$$

Proof. For all $k = 0, \dots, m-1$, let us define

$$V_k(x_k) = \operatorname*{E}_{\varsigma_{k+1}} \left[\sum_{i=k}^{m-1} J_i(x_i, x_{i+1}) \right]. \tag{S6}$$

By applying dynamic programming [6] to problem (7) of the main manuscript, we know that the optimal cost J^* is equal to $V_0^*(x_0)$, obtained as the last step of the recursive algorithm

$$\begin{cases} V_{m-1}^*(x_{m-1}) = J_{m-1}(x_{m-1}, x_m), \\ V_k^*(x_k) = \min\limits_{x_{k+1}} \operatorname*{E}\limits_{\varsigma_{k+1}} \left[J_k(x_k, x_{k+1}) + V_{k+1}^*(x_{k+1}) \right], \qquad k = 0, \dots, m-2. \end{cases} \tag{S7}$$

Now, let us pick any $h \in \{1, \ldots, m-1\}$. If we could write

$$V_h^*(x_h) = \mathop{\mathrm{E}}_{\varsigma_{h+1}} \left[\sum_{i=h}^{m-1} \left(F_h^i x_m - R_h^i x_h \right)^T W_i^{-1} \left(F_h^i x_m - R_h^i x_h \right) \right], \tag{S8}$$

we would then have that

1. *Equation (S2) would hold for $k = h$.* Indeed,

$$\begin{aligned} V_{h-1}^*(x_{h-1}) = &\min_{\substack{x_h \\ \dots \\ x_{m-1}}} \mathop{\mathrm{E}}_{\varsigma_h} \left[\left(x_h - e^{\delta_{h-1} A_{\sigma(h-1)}} x_{h-1} \right)^T W_{h-1}^{-1} \left(x_h - e^{\delta_{h-1} A_{\sigma(h-1)}} x_{h-1} \right) + \sum_{i=h}^{m-1} J_k(x_k, x_{k+1}) \right] \\ = &\min_{x_h} \mathop{\mathrm{E}}_{\varsigma_h} \left[\left(x_h - e^{\delta_{h-1} A_{\sigma(h-1)}} x_{h-1} \right)^T W_{h-1}^{-1} \left(x_h - e^{\delta_{h-1} A_{\sigma(h-1)}} x_{h-1} \right) + V_h^*(x_h) \right] \end{aligned}$$

From (S8), we get

$$\begin{aligned} V_{h-1}^*(x_{h-1}) = \min_{x_h} \Bigg(&\left(x_h - e^{\delta_{h-1} A_{\sigma(h-1)}} x_{h-1} \right)^T W_{h-1}^{-1} \left(x_h - e^{\delta_{h-1} A_{\sigma(h-1)}} x_{h-1} \right) \\ &+ \mathop{\mathrm{E}}_{\varsigma_h} \left[\sum_{i=h}^{m-1} \left(F_h^i x_m - R_h^i x_h \right)^T W_i^{-1} \left(F_h^i x_m - R_h^i x_h \right) \right] \Bigg). \end{aligned} \tag{S9}$$

As the cost function (S9) is convex with respect to x_h, to find its minimum we can compute the gradient and set it to zero, thus obtaining

$$\begin{aligned} &2x_h^T W_{h-1}^{-1} - 2x_{h-1}^T e^{\delta_{h-1} A_{\sigma(h-1)}^T} W_{h-1}^{-1} + 2x_h^T \sum_{i=h}^{m-1} \mathop{\mathrm{E}}_{\varsigma_h} \left[{R_h^i}^T W_i^{-1} R_h^i \right] \\ &- 2x_m^T \sum_{i=h}^{m-1} \mathop{\mathrm{E}}_{\varsigma_h} \left[{F_h^i}^T W_i^{-1} R_h^i \right] = 0, \end{aligned}$$

which implies

$$\begin{aligned} \left(W_{h-1}^{-1} + \sum_{i=h}^{m-1} \mathop{\mathrm{E}}_{\varsigma_h} \left[{R_h^i}^T W_i^{-1} R_h^i \right] \right) x_h = &\sum_{i=h}^{m-1} \mathop{\mathrm{E}}_{\varsigma_h} \left[{R_h^i}^T W_i^{-1} F_h^i \right] x_m \\ &+ W_{h-1}^{-1} e^{\delta_{h-1} A_{\sigma(h-1)}} x_{h-1}. \end{aligned}$$

As matrix $\left(W_{h-1}^{-1} + \sum_{i=h}^{m-1} \operatorname*{E}_{\varsigma_h}\left[{R_h^i}^T W_i^{-1} R_h^i\right]\right)$ is positive definite, we finally get

$$
\begin{aligned}
x_h^* &= \left(W_{h-1}^{-1} + \sum_{i=h}^{m-1} \operatorname*{E}_{\varsigma_h}\left[{R_h^i}^T W_i^{-1} R_h^i\right]\right)^{-1} \sum_{i=h}^{m-1} \operatorname*{E}_{\varsigma_h}\left[{R_h^i}^T W_i^{-1} F_h^i\right] x_m \\
&+ \left(W_{h-1}^{-1} + \sum_{i=h}^{m-1} \operatorname*{E}_{\varsigma_h}\left[{R_h^i}^T W_i^{-1} R_h^i\right]\right)^{-1} W_{h-1}^{-1} e^{\delta_{h-1} A_{\sigma(h-1)}} x_{h-1} \\
&= P_h x_m + Q_h x_{h-1}.
\end{aligned}
\tag{S10}
$$

2. *Equation (S8) also holds for $k = h-1$.* Indeed, combining (S9) and (S10), we get

$$
\begin{aligned}
V_{h-1}^*(x_{h-1}) &= \left(P_h x_m + \left(Q_h - e^{\delta_{h-1} A_{\sigma(h-1)}}\right) x_{h-1}\right)^T W_{h-1}^{-1} \left(P_h x_m + \left(Q_h - e^{\delta_{h-1} A_{\sigma(h-1)}}\right) x_{h-1}\right) \\
&+ \operatorname*{E}_{\varsigma_h}\left[\sum_{i=h}^{m-1} \left(\left(F_h^i - R_h^i P_h\right) x_m - R_h^i Q_h x_{h-1}\right)^T W_i^{-1} \left(\left(F_h^i - R_h^i P_h\right) x_m - R_h^i Q_h x_{h-1}\right)\right]
\end{aligned}
\tag{S11}
$$

Now, if we set $F_{h-1}^{h-1} = P_h$, $R_{h-1}^{h-1} = e^{\delta_{h-1} A_{\sigma(h-1)}} - Q_h$, $F_{h-1}^i = F_h^i - R_h^i P_h$, and $R_{h-1}^i = R_h^i Q_h$, for all $i = h, \ldots, m-1$, equation (S11) can be rewritten as in (S8), that is,

$$
V_{h-1}^*(x_{h-1}) = \operatorname*{E}_{\varsigma_h}\left[\sum_{i=h-1}^{m-1} \left(F_{h-1}^i x_m - R_{h-1}^i x_{h-1}\right)^T W_i^{-1} \left(F_{h-1}^i x_m - R_{h-1}^i x_{h-1}\right)\right]. \tag{S12}
$$

From (S7), and setting $F_{m-1}^{m-1} = I$ and $R_{m-1}^{m-1} = e^{\delta_{m-1} A_{\sigma(m-1)}}$, we know that (S8) holds for $h = m-1$. By induction, the thesis follows. □

Notice that the above solution can also be applied in a deterministic setting when the sequence of snapshots is known by simply dropping the expected values. In that scenario, the iterative nature of our solution yields a substantial computational advantage compared to [5].

S2 Behavior of the optimal solution

As most real systems in their normal mode of operation exhibit stability [7], we focus on the case in which, for all k, all the admissible topologies $A_{\sigma(k)}$ are described by Hurwitz matrices Under

this hypothesis, we will show that when the network temporality is extremely slow ($\delta \to \infty$), then the difference between expected energy J^*_{temporal} associated to the optimal solution and the energy J^*_{static} associated to the static benchmark tends to be negligible. On the contrary, when the network variability is extremely fast ($\delta \to 0$), temporality becomes detrimental, that is $J^*_{\text{temporal}} > J^*_{\text{static}}$, with the only exception of the two-snapshot case in which $J^*_{\text{temporal}} = J^*_{\text{static}}$.

For ease of illustration, we first derive our results in the two-snapshot case, and $x_0 = 0$. Then, we extend the the derivations to the general case.

S2.1 The case of two snaphots

In this case, the expected optimal energy for controlling the stochastic temporal network can be written as

$$\begin{aligned} J^*_{\text{temporal}} = \underset{\sigma(0)}{\mathrm{E}}\left[J^*\right] &= x_2^T \,\mathrm{E}\left[W_1^{-1}\right] x_2 \\ &- x_2^T \,\mathrm{E}\left[W_1^{-1} e^{\delta A_{\sigma(1)}}\right] \mathrm{E}\left[\left(\mathrm{E}\left[W_{c,1}^{-1}\right] + W_0^{-1}\right)^{-1}\right] \mathrm{E}\left[e^{\delta A^T_{\sigma(1)}} W_1^{-1}\right] x_2, \end{aligned} \tag{S13}$$

where $W_{c,1}$ is the controllability gramian, which is by definition related to the reachability gramian as follows:

$$W_{c,1}^{-1} = e^{\delta A^T_{\sigma(1)}} W_1^{-1} e^{\delta A_{\sigma(1)}}.$$

Taking the limit for $\delta \to +\infty$, as the spectrum of the matrices $A_{\sigma(k)}$ belongs to the open-left half complex plane for all k, we have

$$\lim_{\delta \to +\infty} e^{\delta A_{\sigma(1)}} = 0.$$

Now, taking advantage of the following well-known relationship between the finite-time and infinite-time gramians [8]

$$W_1 = W_1^\infty - e^{\delta A_{\sigma(1)}} W_1^\infty e^{\delta A^T_{\sigma(1)}},$$

where

$$W_1^\infty = \int_{t_0}^{\infty} e^{\tau A_{\sigma(1)}} B B^T e^{\tau A^T_{\sigma(1)}} \mathrm{d}\tau,$$

one obtains

$$\lim_{\delta \to +\infty} W_1 = W_1^{\infty}, \text{ and } \lim_{\delta \to +\infty} W_{c,1}^{-1} = 0,$$

thus finally yielding

$$\lim_{\delta \to +\infty} J^*_{\text{temporal}} = x_2^T \operatorname{E}\left[(W_1^{\infty})^{-1}\right] x_2 = \lim_{\delta \to +\infty} J^*_{\text{static}}. \tag{S14}$$

A similar result can be achieved when the network variability is much faster than the fastest time constant of all possible snapshots. Indeed, when $\delta \to 0$, one obtains

$$e^{\delta A_{\sigma(k)}} = I_n, \tag{S15}$$

for all $k = 0, \ldots, m-1$, and for all $\sigma(k)$ associated to a positive value of the probability density function $f(\sigma(k))$. This also implies that

$$\lim_{\delta \to 0} W_{c,k} = \lim_{\delta \to 0} W_k = \lim_{\delta \to 0} \delta B B^T \tag{S16}$$

independently from the realization of $\sigma(k)$. Hence, from (S13), and leveraging the Moore-Penrose pseudo-inverse as BB^T might not be invertible, we get

$$\begin{aligned} \lim_{\delta \to 0} J^*_{\text{temporal}} &= \lim_{\delta \to 0} \left(x_2^T \frac{(BB^T)^\dagger}{\delta} x_2 - x_2^T \frac{(BB^T)^\dagger}{\delta} \frac{\delta}{2} B B^T \frac{(BB^T)^\dagger}{\delta} x_2 \right) \\ &= \lim_{\delta \to 0} \frac{1}{2} x_2^T \frac{(BB^T)^\dagger}{\delta} x_2 \end{aligned}, \tag{S17}$$

which is exactly what we obtain also for the static benchmark, as we indeed have

$$\lim_{\delta \to 0} \operatorname*{E}_{\sigma}\left[W_\sigma\right] = \lim_{\delta \to 0} 2\delta B B^T.$$

Hence, leveraging again the Moore-Penrose pseudo-inverse as BB^T might not be invertible, we get

$$\lim_{\delta \to 0} J^*_{\text{static}} = \lim_{\delta \to 0} x_m^T W_\sigma^{-1} x_m = \lim_{\delta \to 0} x_m^T \frac{(BB^T)^\dagger}{2\delta} x_m = \lim_{\delta \to 0} J^*_{\text{temporal}}.$$

Note that the use of the Moore-Penrose pseudo-inverse admits a very simple interpretation. Both in the static and in the temporal case, when $\delta \to 0$, the reachable subspace shrinks to the

column space of BB^T (which is nothing but the column space of B), and reaching the states in this subspace requires infinite energy.

S2.2 Generalization

Case $\delta \to 0$

When $\delta \to 0$, from the recursive equations (S3) and (S4), some algebra allows to derive that

$$P_k, Q_k = \frac{I_n}{2}, \qquad k = 1, \ldots, m-1, \tag{S18}$$

and, for all $k = 0, \ldots, m-1$, that

$$F_k^i = R_k^i = \alpha_k^i I_n, \qquad 0 \leq k \leq i \leq m-1, \tag{S19}$$

where

$$\alpha_k^i = \begin{cases} 1, & i = k, i = m-1, \\ 1/2, & i = k, i < m-1, \\ \alpha_k^{i-1}/2, & i > k, i < m-1, \\ \alpha_k^{i-1}, & i > k, i = m-1. \end{cases} \tag{S20}$$

Notice that as $\sum_{i=k}^{m-1} \alpha_k^i = 1$ and $\alpha_k^i > 0$ for all i, k, then

$$\sum_{i=k}^{m-1} {\alpha_k^i}^2 \geq \frac{1}{m-k}, \qquad k = 0, \ldots, m-1. \tag{S21}$$

From (S5), and leveraging again the Moore-Penrose pseudo-inverse, we can then write

$$\lim_{\delta \to 0} \operatorname*{E}_{\sigma(0)} [J^*] = \sum_{i=0}^{m-1} {\alpha_0^i}^2 \lim_{\delta \to 0} (x_m - x_0)^T \frac{(BB^T)^\dagger}{2\delta} (x_m - x_0). \tag{S22}$$

On the other hand, the static benchmark when δ tends to zero becomes

$$\lim_{\delta \to 0} \operatorname*{E}_{\sigma} [J_{\mathrm{s}}^*(\sigma)] = \frac{1}{m} \lim_{\delta \to 0} (x_m - x_0)^T \frac{(BB^T)^\dagger}{2\delta} (x_m - x_0). \tag{S23}$$

Now, from (S21) we have that $\sum_{i=0}^{m-1} {\alpha_0^i}^2 \geq 1/m$. Considering that $\sum_{i=0}^{m-1} {\alpha_0^i}^2 = 1/m$ if and only if $\alpha_0^i = \alpha_0^j$ for all i, j, we can finally conclude that

$$\lim_{\delta \to 0} \operatorname*{E}_{\sigma(0)} [J^*] \begin{cases} = \lim\limits_{\delta \to 0} \operatorname*{E}\limits_{\sigma} [J_{\rm s}^*(\sigma)] & \text{if } m = 1, 2, \\ > \lim\limits_{\delta \to 0} \operatorname*{E}\limits_{\sigma} [J_{\rm s}^*(\sigma)] & \text{if } m \geq 3. \end{cases} \tag{S24}$$

Moreover, by combining equations (S2) and (S18), we obtain

$$x_k^* = \frac{x_{k-1} + x_m}{2}, \tag{S25}$$

that is, the next optimal waypoint is halfway between the current waypoint and the final state x_m.

Case $\delta \to +\infty$

When $\delta \to +\infty$, we have that $\lim_{\delta \to +\infty} e^{\delta A_{\sigma(k)}} = 0$ for all possible realization of $\sigma(k)$, and for all k. Furthermore,

$$\lim_{\delta \to +\infty} W_k = W_k^\infty := \int_{t_0}^{+\infty} e^{\tau A_{\sigma(k)}} B B^T e^{\tau A_{\sigma(k)}^T} \mathrm{d}\tau.$$

Now, notice that from equations (S3) and (S4) we can write

$$\begin{aligned} R_k^i &= 0_n, \quad k = 0, \dots, m-1, \ k \leq i \leq m-1 \\ F_k^i &= \begin{cases} I_n, & \text{if } i = m-1, \\ 0_n, & \text{otherwise,} \end{cases} \end{aligned} \tag{S26}$$

This yields

$$\lim_{\delta \to +\infty} \operatorname*{E}_{\sigma(0)} [J^*] = x_m^T \operatorname*{E}_{\sigma(m-1)} \left[(W_{m-1}^\infty)^{-1} \right] x_m. \tag{S27}$$

Now, observing that $\operatorname*{E}_{\sigma(m-1)} \left[(W_{m-1}^\infty)^{-1} \right] = \lim_{\delta \to +\infty} \operatorname*{E}_{\sigma} \left[W_\sigma^{-1} \right]$, we finally get

$$\lim_{\delta \to +\infty} \operatorname*{E}_{\sigma(0)} [J^*] = \lim_{\delta \to +\infty} \operatorname*{E}_{\sigma} [J_{\rm s}^*(\sigma)]. \tag{S28}$$

Notice that equations (S3) and (S4) also implies that

$$P_k = 0_n, \quad Q_k = 0_n, \quad k = 1, \dots, m-1, \tag{S29}$$

thus yielding $x_k^* = 0$ for all $k = 1, \dots, m-1$.

S3 Description of the empirical dataset

To show the effectiveness of our approach in controlling temporal networks in the stochastic setting, we perform numerical simulations both on synthetic and empirical data set.

Empirical data set

Protein network: The raw data set is the time series of gene expression (GSE4987) coming from GEO (Gene Expression Omnibus) repository and consists of 6228 probes at 50 different time points [9]. To reconstruct the temporal network, we filter the data employing the method presented in [10]. Namely,

1) At each time point $t \in \{1, \ldots, 50\}$, we compute the activity level $\mathrm{act}_i(t)$ of the i-th gene, for all $i = 1, \ldots, 6228$, following [10];

2) We compare $\mathrm{act}_i(t)$ with a global activity threshold τ for all the genes. At each snapshot, we say that there is an undirected edge between nodes j and h if $\mathrm{act}_i(t) > \tau$ for $i = j, h$.

3) Finally, according to the gene ontology terms, we consider a reduced network obtained by considering only the genes sharing the same *Biological Process*.

As discussed in Section S2, although our optimal solution also works for unstable dynamics, we focus on networks associated to stable (dissipative) dynamics. Therefore, we add suitable self-loops so as to make the adjacency matrix Hurwitz in all the snapshots.

Synthetic data set

To perform our analyses on synthetic temporal networks, we build a pool of three ER-like undirected graphs with average degree 6 and $n = 100$ nodes. The edge weights of each graphs are randomly selected in the interval $(0, 1]$. Their adjacency matrices are manipulated so as to obtain laplacian matrices.

For the numerical analysis portrayed in Figure 2 of the main text, we build the three snapshots starting from the obtained three laplacian matrices stabilizing their standalone dynamics according to the Gershgorin disks theorem, that is, adding to their diagonal elements the scalars $\{-3, -1, -2\}$.

For the numerical analysis of Figure 3 of the main text, we start from the same set of three laplacians. From these matrices we create 19 pools of 3 snapshots stabilizing their standalone dynamics so that each matrix in the same pool shares the same maximum eigenvalue $\lambda_{\max}$, each pool being characterized by different $\lambda_{\max}$. The maximum eigenvalues selected for each pool are

$$\{-20, -18, -16, -13, -12, -10, -8, -6, -4, -2$$
$$-1.8, -1.6, -1.4, -1.2, -1, -0.8, -0.6, -0.4, -0.2\}.$$

In this way, the dynamics associated to each pool are characterized by increasing dominant time constants $\tau_{\max} = 1/|\lambda_{\max}|$.

For all numerical analyses performed on synthetic networks (Figures 2 and 3 of the main text), each snapshot of a temporal network is extracted from its pool according to a uniform distribution. Moreover, a common set of 10 driver nodes has been selected so to ensure controllability of each snapshot of all the pools.

S4 Additional Numerical Results

In this section, we present the results of three additional sets of simulations aiming at testing the robustness of our findings to the removal of the assumption of independence between the variables of the stochastic process $\sigma(k)$, to the size of the pool $\mathcal{F}$, and to an increase of the number of snapshots m. In the first set of simulations, we remove the assumption on the stochastic process $\sigma(k)$ being i.i.d., and model the dependence between consecutive snapshots through a Markov Chain. Specifically, the transition probability matrix between the three possible net-

work topologies has been selected as

$$\Pi = \begin{bmatrix} 0.3 - 0.05s & 0.4 + 0.1s & 0.3 - 0.05s \\ 0.3 - 0.05s & 0.3 - 0.05s & 0.4 + 0.1s \\ 0.4 + 0.1s & 0.3 - 0.05s & 0.3 - 0.05s \end{bmatrix}, \tag{S30}$$

for all $s = 0, 1, \ldots, 5$. Note that, for any value of s, we have that the a posteriori probability is $\Pr\{A_{\sigma(k)} = F_i | A_{\sigma(k-1)} = F_j\} = 0.3 + 0.1s$ if $(i, j) \in \{(2, 1), (3, 2), (1, 3)\}$ and $\Pr\{A_{\sigma(k)} = F_i | A_{\sigma(k-1)} = F_j\} = 0.3 - 0.05s$ otherwise, while the a priori probability is $\Pr\{A_{\sigma(k)} = F_i\} = 1/3\ \forall i, j, k$. Hence, the Markov Chain ensures that $\Pr\{A_{\sigma(k)} = F_i | A_{(k-1)} = F_j\} \neq \Pr\{A_{\sigma(k)} = F_i\}$, thereby introducing a dependence between the variables of the stochastic process $A_{\sigma(k)}$. Finally, note that, as s increases, the process $A_{\sigma(k)}$ becomes more and more predictable with the limit case, not of interest for this work, being $s = 6$ where stochasticity vanishes and the sequence of snapshots becomes deterministic. Consistently, Figure S1 shows that when s increases the advantage of temporal over static networks increases, but again, only if the network temporality matches the time-scale of the nodes we want to control.

In the second set of simulations, we test the robustness of our findings to an increase in the size of the pool $\mathcal{F}$. Namely, we build a pool $\mathcal{F}_{\text{ER}}$ of five ER-like undirected graphs constructed as in Section S3. Then, we compare the expected energy required to control a three-snapshot temporal network when we consider the finite set of all the possible pools $\mathcal{F}_1, \ldots, \mathcal{F}_{10}$ of cardinality $|\mathcal{I}| = 3$ extracted from $\mathcal{F}_{\text{ER}}$ against the case in which the pool is $\mathcal{F}_{\text{ER}}$ itself.

Consistently with the result presented in the main text, we observe that uncertainty still prevails of temporality in the fast regime by an even wider margin, and that temporality prevails when it matches the time scale of the nodes we want to control.

In the third and final set of additional simulations, we test the robustness of our results to increasing the number of snapshots m. Namely, we extend the analysis presented for $m = 3$ and $|\mathcal{I}| = 5$ to the case of $m = 4$ and $m = 5$. Before discussing the outcome of this additional numerical analysis, let us note that when m is increased an additional degree of freedom is

given to the control designer, and thus the control energy can only decrease. Indeed, when $m > 3$, selecting $x(t_k) = 0$ for all $k < m - 2$ and then using the solution obtained for the case of $m = 3$ would yield the same control energy of the case of three snapshots. Consistently, Fig. S3 illustrates that as m increases the control energy decreases. However, the energy gains are relevant mostly when δ is small, that is, mostly when the control horizon $[t_0,\ t_m]$ is such that control becomes energetically prohibitive. Summing up, when the number of snaphsots increases, exploiting temporality to our advantage is possible for a slightly wider interval of values of δ.

References

[1] P. Holme and J. Saramäki, “Temporal networks,” *Physics Reports*, vol. 519, no. 3, pp. 97–125, 2012.

[2] T. Spyropoulos, K. Psounis, and C. S. Raghavendra, “Spray and wait: an efficient routing scheme for intermittently connected mobile networks,” in *Proceedings of the 2005 ACM SIGCOMM workshop on Delay-tolerant networking*, 2005, pp. 252–259.

[3] D. S. Bassett, N. F. Wymbs, M. A. Porter, P. J. Mucha, J. M. Carlson, and S. T. Grafton, “Dynamic reconfiguration of human brain networks during learning,” *Proceedings of the National Academy of Sciences*, vol. 108, no. 18, pp. 7641–7646, 2011.

[4] J.-P. Onnela, A. Chakraborti, K. Kaski, J. Kert’esz, and A. Kanto, “Dynamics of market correlations: Taxonomy and portfolio analysis,” *Physical Review E*, vol. 68, no. 056110, pp. 1–12, 2003.

[5] A. Li, S. P. Cornelius, Y.-Y. Liu, L. Wang, and A.-L. Barabási, “The fundamental advantages of temporal networks,” *Science*, vol. 358, no. 6366, pp. 1042–1046, 2017.

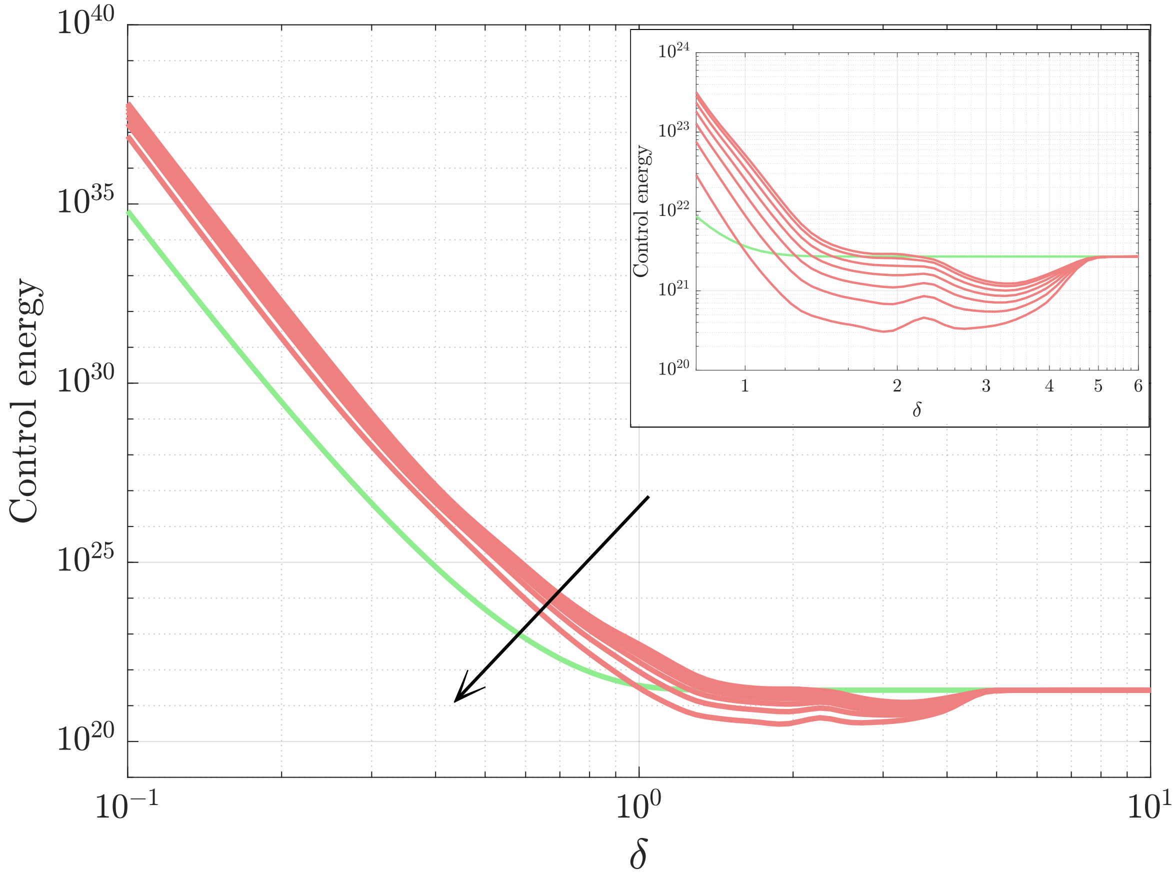

Figure S1: **Effect of introducing a dependence between consecutive snapshots.** The red lines identify the expected energy required to control the network for different values of the parameter s in (S30). The black arrow points towards increasing values of s, which is varied between 0 and 5 with step 1. The green line identifies the static benchmark.

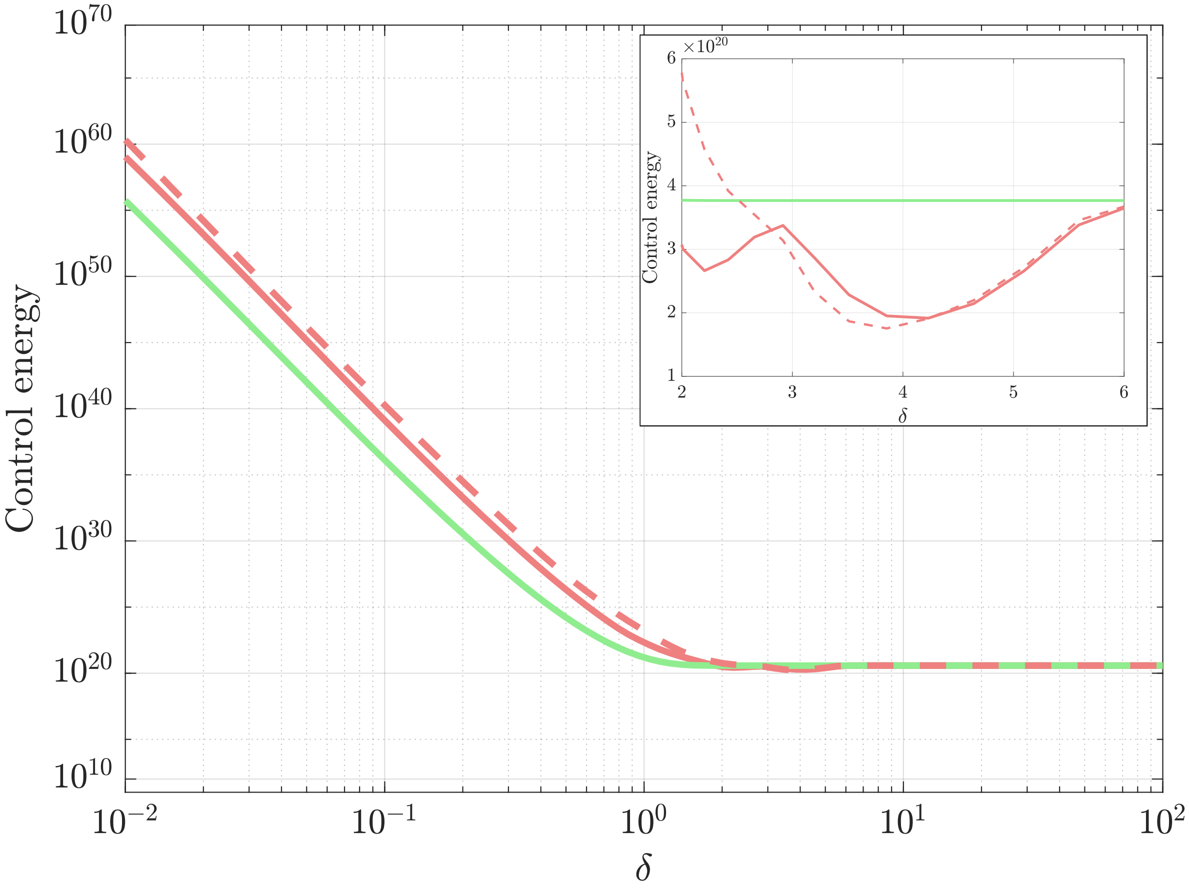

Figure S2: **Effect of the size of the pool $\mathcal{F}$.** The red dashed line identifies the expected energy required to control the network when the pool of five ER-like topologies $\mathcal{F}_{\mathrm{ER}}$ is considered, while the red solid line corresponds to the expected energy averaged over all the possible pools $\mathcal{F}_1, \ldots, \mathcal{F}_{10}$ of cardinality $|\mathcal{I}| = 3$ that can be extracted from $\mathcal{F}_{\mathrm{ER}}$. The green solid line identifies the static benchmark when the pool is $\mathcal{F}_{\mathrm{ER}}$.

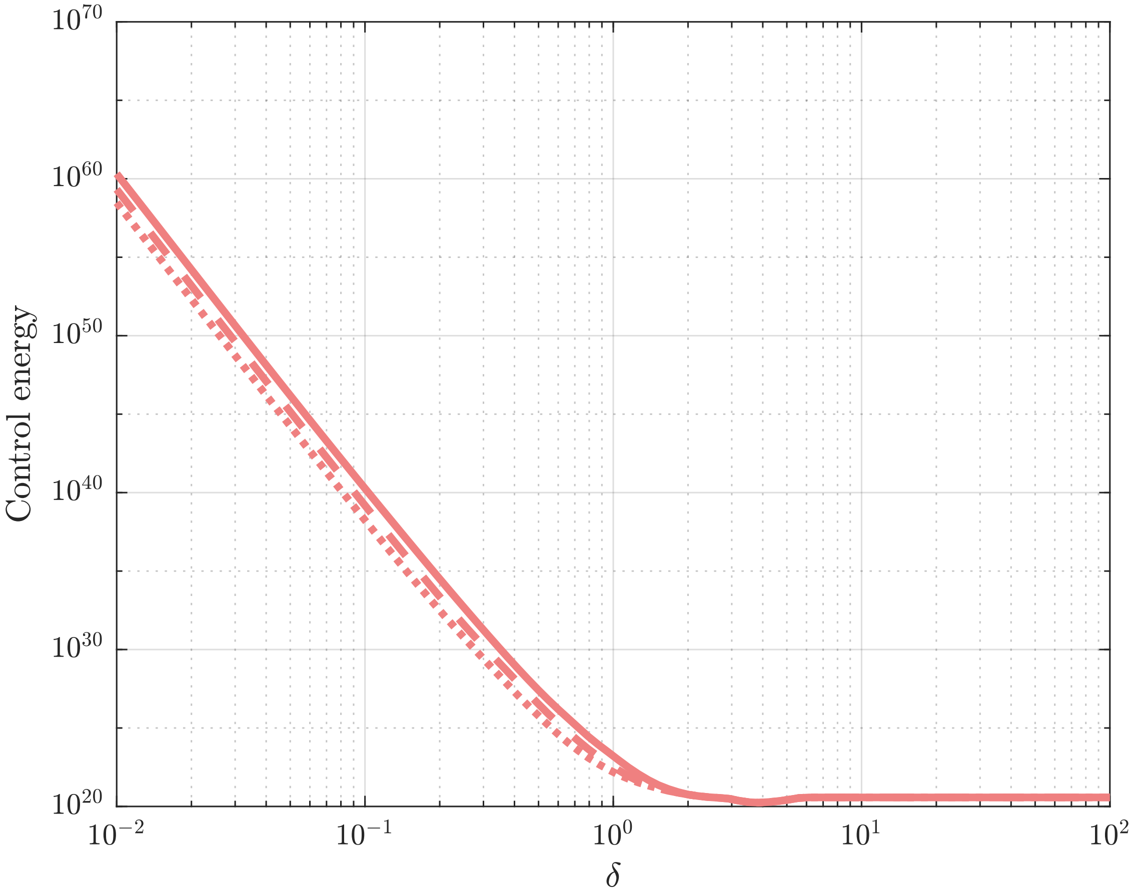

Figure S3: **Effect of increasing the number of snapshots m.** The red solid, dashed, and dotted lines identify the expected energy required to control the network when m is equal to 3, 4, and 5 respectively.

[6] R. Bellman, "Dynamic programming," *Science*, vol. 153, no. 3731, pp. 34–37, 1966.

[7] R. M. May, *Stability and Complexity in Model Ecosystems*. Princeton, NJ, USA: Princeton University Press, 1973.

[8] W. Gawronski and J.-N. Juang, "Model reduction in limited time and frequency intervals," *International Journal of System Science*, vol. 21, no. 2, pp. 349–376, 1990.

[9] "https://www.ncbi.nlm.nih.gov/geo/query/acc.cgi?acc=gse4987."

[10] J. Wang, X. Peng, M. Li, and Y. Pan, "Construction and application of dynamic protein interaction network based on time course gene expression data," *Proteomics*, vol. 13, no. 2, pp. 301–312, 2013.
